# Screening and combination selection of multifunctional actinomycetes for aerobic composting

**DOI:** 10.1128/spectrum.02053-23

**Published:** 2023-10-31

**Authors:** Zixiao Zhao, Huanzhang Liu, Suxing Zhao, Huiqia Liu, Yuer Han, Jianchai Liu

**Affiliations:** 1 College of Life Science and Food Engineering, Hebei University of Engineering, Handan, China; 2 College of Landscape and Ecological Engineering, Hebei University of Engineering, Handan, China; 3 Emergency Department of Prevention and Control, Handan Animal Disease Prevention and Control Center, Handan, China; 4 Agricultural Service Centre, Gaoyi County Fucun Town People’s Government, Shijiazhuang, China; Center of Innovative and Applied Bioprocessing, Mohali, Punjab, India

**Keywords:** aerobic composting, actinomycetes, screening, combination of strains

## Abstract

To screen the most effective combination of multipurpose actinomycete strains for aerobic composting, lignocellulose-degrading strains were obtained through enrichment culture, separation and purification, and multiselection culture. The isolated strains were identified on the basis of morphology and 16S rRNA gene sequencing and comparison. The obtained actinomycetes were screened for the lignocellulose degradation rate, ammonia and hydrogen sulfide removal rate, and cellulase activity. Subsequently, a combination of strains with ecological coordination and complementary functions was determined through antagonistic tests. The combined strains were subjected to a straw fermentation test to evaluate their degradation effect. As a result, 20 strains of actinomycetes were isolated and identified from more than 56 samples from different sources, including 18 strains of *Streptomyces* sp. and 2 strains of *Nocardiopsis* sp. After a number of screenings, eight strains of actinomycetes with strong function were obtained. From the results of function and antagonistic tests, strains A3, A7, A8, A11, and A16 were selected to form consortia. The straw fermentation test showed that the lignocellulose degradation rate of the strain combination was as high as 39.8%, which was significantly higher than that of eachevery single strain (*P* < 0.05). The screened multifunctional actinomycete strains and their combination enriched the actinomycete germplasm resources used for microbial enhanced aerobic composting.

**IMPORTANCE:**

With the development of animal husbandry in China, the production of a large amount of livestock and poultry manure has become one of the main agricultural pollution sources. High-temperature aerobic composting stands out as one of the most crucial methods for the safe and resourceful utilization of livestock and poultry manure, serving as an essential link between crop cultivation, animal breeding, and sustainable agricultural development. Numerous studies have demonstrated that the addition of exogenous multifunctional bacterial agents to compost reduces not only harmful emissions but also sequesters or increases essential nutrients. However, these efficacies depend on the specific functions of the bacteriophage itself, the harmonization and complementarity within the colony, and its ability to adapt to the environment. In recent years, relatively few studies have been conducted on actinomycetes. This experiment provides excellent actinomycete resources for the production of high-efficiency and high-quality compost compound microbial agents of manure and straw.

## INTRODUCTION

China is a large agricultural country; with the development of animal husbandry, a large amount of breeding manure has also brought huge environmental problems, and even the safety of animal products and other hazards to animal and human health (1 – 3). Therefore, livestock and poultry manure should take the necessary measures to degrade the harmful substances and harmful gases before use (4 – 6). This experiment intends to enrich, isolate, and screen multi-functional actinomycetes strains suitable for high-temperature aerobic composting from a variety of natural and man-made environmental samples. Through the ecological relationship test between strains, according to the principles of ecological coordination, functional complementarity and appropriate number of strains, the combination of strains is scientifically selected to provide valuable strain resources for the selection and application of fecal aerobic composting composite microbial agents.

## MATERIALS AND METHODS

### Medium

Lignocellulosic, Gao’s No. 1, cellulose Congo red, lignin aniline blue, lignin bright blue, straw degradation, NH_3_ selective, autotrophic nitrification, heterotrophic nitrification, Ashube nitrogen-free, H_2_S selective, PDA composite, and GY liquid media were made in our laboratory.

### Sample collection

A total of 56 samples of livestock manure, compost, mushroom fermentation material, humus layer, and field soil were collected from livestock farm, organic fertilizer plant, edible mushroom farm, woodland, and farmland in the Handan area. The samples were labeled by category, sampling point, and sampling time and brought back to the laboratory on the same day.

### Enrichment and separation

All samples were classified and mixed in accordance with the collection area. Twenty grams of each sample was mixed thoroughly and then divided into two portions. Each portion was placed into a sterilized 250 mL conical flask containing 100 mL of a lignocellulose-enriched culture solution. The flasks were shaken at 35°C and 45°C at 160 r/min for 48 hr.

The enriched culture was fully shaken, from which 1.0 mL of the suspension was taken and placed in a test tube containing 9 mL of sterile water, and a 10^−1^ dilution was obtained after shaking. Through this method, 10^−2^–10^−5^ dilutions were obtained. BThe bacterial suspension (100 µL) of enriched cultures of all dilutions was drawn and evenly spread on the culture dish of Gao’s No. 1 medium; this process was repeated two times. The Petri plates were incubated at 35°C and 45°C for 1–7 days. During the culture period, the colony growth was observed every day, and different single colonies with round, flat, or many wrinkled suspected actinomycetes were selected from the appropriate dilution plate. They were purified and cultured on the new Gao’s No. 1 medium plate. The streak culture was repeated to obtain pure strains.

### Strain identification

#### Morphological observation

The isolated strains were inoculated in Gao’s No. 1 Petri plate by the continuous streaking method and incubated at 35°C for 2–7 days. The colonial growth and morphological characteristics were observed every day. The mycelium in a single colony was extracted with an inoculation loop at an appropriate time, Gram staining was performed, and the mycelial morphology was observed with a microscope. The classification of strains was preliminarily judged in accordance with the morphology of the colony and mycelium.

#### Molecular identification

The genomic DNA of the isolated strains was extracted via the cetyltrimethylammonium bromide (CTAB)-SDS method (7). The extracted genomic DNA was amplified with universal primers of a prokaryotic 16S rDNA full-length sequence. The primer sequence was 27F: 5′-AGAGTTTGATCCTGGCTCAG-3′,1492R:5′-GGTTACCTTGTTACGACTT-3′, and the amplified products were sent to Senggong Bioengineering (Shanghai) Co., Ltd. for sequencing. The sequencing results were compared with the nucleic acid sequences in the NCBI database by BLAST to find the strains with the highest similarity. The species of strains were determined by morphological observation and molecular identification.

### Preliminary screening of the isolates

The isolated strains were sequentially subjected to a series of functional tests through the following methods: (i) Cellulose-degrading microbes were screened by a cellulose Congo red selective culture method (8). (ii) Lignin-degrading microbes were screened by lignin aniline blue and brilliant blue selective culture (9). (iii) Ammonia assimilation microbes were screened by an ammonia nitrogen selective culture method (10, 11). (iv) Sulfur-oxidizing microbes were screened by a hydrogen sulfide selective culture method (12). (v) Nitrifying microbes were screened by a nitrite nitrogen selective culture method (13, 14). (vi) Nitrogen-fixing microbes were screened by a nitrogen-free medium selection method (15). (vii) Thermotolerant microbes were screened by a high-temperature (60°C, 30 min) pretreatment method (16, 17).

### Functional rescreening

The strains capable of cellulose degradation, lignin degradation, ammonia assimilation, and vulcanization were rescreened. On the basis of the lignocellulose degradation rate, cellulase activity, and ammonia and hydrogen sulfide removal rate, combined with the results of the preliminary screening, the candidate strains for strain combination were initially selected.

#### Determination of lignocellulose degradation rate

The degradation rate for lignocellulose was determined by a weight loss method (18). The specific method was as follows: Degradation rate (%) = (lignocellulose mass in substrate before inoculation − lignocellulose mass in substrate after culture) / lignocellulose mass in substrate before inoculation ×100%.

#### Determination of cellulase enzyme activity

The specific method, i.e., the 3,5-Dinitrosalicylic acid (DNS) method, is shown in He Songjie and Yong’s article (19). The CMC enzyme activity unit:, Tthe amount of enzyme required to hydrolyze sodium carboxymethyl cellulose into 1.0 µg of glucose per minute, was defined as one enzyme activity unit U (namely, IU/mL).

#### Detection of ammonia and hydrogen sulfide

A boric acid absorption method and zinc ammonium complex salt spectrophotometry were used (20). That is, ammonia (hydrogen sulfide) removal rate (%) = (control value − treatment value) / control value ×100%.

### Selection of strain combination

#### Antagonism test

The candidate strains were subjected to a Petri plate confrontation test in pairs by using Gao’s No. 1 medium (21) . The strains were enriched in a liquid medium, and 100 µL of bacterial suspension was drawn, plated on the medium, and cultured at 35°C for 3 days. A circular puncher was used to punch in the solid medium, and the strains were attached to the new Gao’s No. 1 solid medium in pairs. The strains were cultured at 35°C for 5–7 days, and whether the two strains could coexist harmoniously was observed in time. The strains with antagonistic relationship were determined and recorded.

#### Combination matching

The rescreened strains were evaluated in accordance with the category and intensity of the function, and the sum of multifunctional scores of each strain was calculated. The strains were sorted in the order of the scores, and the primary screening functions of the strains with the same or similar scores could be compared. On the basis of the principle of full function, high score, no antagonism, and appropriate number of strains, the expected better strain combination was selected.

### Functional test of strain combination

#### Preparation of complex microbial inoculant

The single strain cultures from the combination were inoculated in Gao’s No. 1 agar Petri plate with sterile cotton swabs and incubated at 35°C for 7–10 days. A microbial cake was taken from the plate by using a sterile punch with a diameter of 6 mm, inoculated into a shake flask with 200 mL of a GY liquid medium, and incubated at 35°C for 3–5 days, three parallel. After cultures with no pollution were identified and confirmed, 20 mL of culture was extracted from each flask and transferred to another sterile flask. The flask was then vigorously shaken for later use.

#### Test of straw fermentation

With reference to the method of 1.6.1 (18), the single strain culture and complex microbial inoculant of the combination were inoculated into a straw fermentation medium at 3% inoculation rate and shaken at 30°C at 160 r/min for 5 days. The straw fermentation test of each strain was performed with three replicates. The straw degradation rate of each treatment was measured at the end of fermentation. The data were analyzed using SPSS 20.0 statistical software. A significance level of *P* < 0.05 was considered to determine statistical significance.

## RESULTS

### Separation and preliminary screening

Through enrichment culture, strain separation, and purification of the samples, 20 suspected actinomycete strains were obtained, and the isolates were subjected to multiple rounds of functional screening. The results are shown in Table 1.

**TABLE 1:** The isolated strains and their primary screening results^*a*^

Strain number	Cellulose degradation (mm)	Lignin degradation (mm)	Ammonia assimilation	Vulcanization	Nitrification	Nitrogen fixation	Heat resistance (60°C)
A1	5.64	0.4	±	−	−	+	−
A2	12.62	0.7	+	+	−	−	−
A3	14.73	1.2	+++	++	+	−	++
A4	15.50	1.5	++++	++	−	−	−
A5	17.03	1.4	++++	++	−	−	−
A6	16.51	0.5	+++	+	−	−	−
A7	15.17	4.2	+++++	+++	−	+++	+++
A8	14.87	2.3	++++	++	−	++	−
A9	16.02	0.4	++++	++	−	±	−
A10	5.68	0.6	++	++	−	−	+++
A11	13.03	5.1	++++	+++	+	+++	+++
A12	4.69	0.7	++	+	−	+	++
A13	7.35	0.2	+	++	−	+	++
A14	6.61	0.9	+	+	−	−	+
A15	6.38	0.4	−	±	−	−	+
A16	17.07	3.3	++++	+++	±	+++	+++
A17	5.42	0.5	++	++	±	+	+
A18	5.02	0.6	++	++	−	+	+
A19	17.60	5.6	+++	++	++	−	−
A20	4.03	0.2	±	−	+	−	−

^*a*^ −, did not have the function; ±, had the function but not significant; +, had the function. The number of + symbols indicates the relative strength of the function. Functions of ammonia assimilation, vulcanization, nitrification, nitrogen fixation, and heat resistance were indicated according to the medium turbidity,: slight turbidity recorded as +, mild as ++, moderate as +++, severe as ++++, and high as +++++.

**Fig 1:**
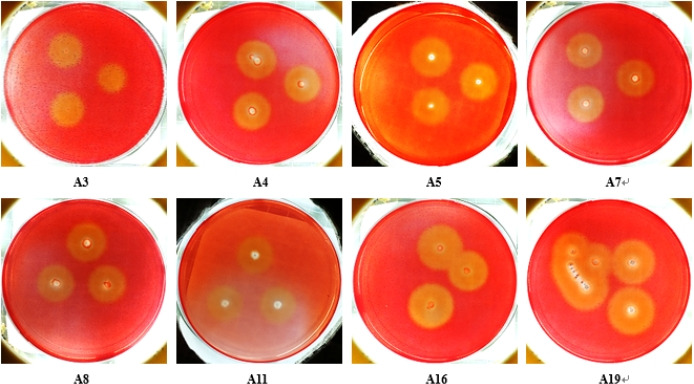
Colony and degradation cycle of some strains screened on cellulose Congo red medium.

**Fig 2:**
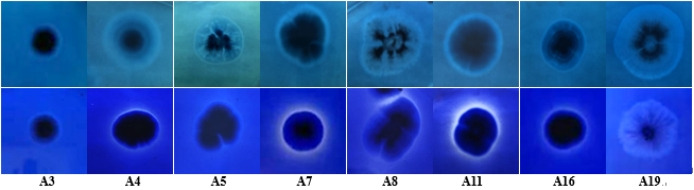
Growth of strains screened on medium of lignin aniline blue (top) and lignin bright blue (bottom).

In accordance with the preliminary screening results, eight strains with more and stronger functions were selected. Their growth and degradation in cellulose Congo red medium, lignin aniline blue medium, and lignin bright blue medium Petri plates are shown in Fig. 1 and 2, respectively.

### Rescreening

The strains with obvious effects of cellulose and lignin degradation, ammonia assimilation, and vulcanization in the primary screening were rescreened. The effect values and aggregate scores of the strains are shown in Table 2.

**TABLE 2:** Effect values and aggregate score of rescreening strains

Strain number	Lignocellulose degradation rate (%)	CMCase (U)	Ammonia removal rate (%)	Hydrogen sulfide removal rate (%)	Aggregate scores
A3	35.1	42.50 ± 1.30	34.3	26.8	140.0
A4	31.2	32.35 ± 0.75	45.9	12.1	122.3
A5	29.8	29.30 ± 1.11	42.7	28.7	131.6
A6	27.5	25.89 ± 0.81	33.2	12.5	99.9
A7	32.6	35.19 ± 1.11	58.5	33.1	160.5
A8	30.2	22.37 ± 0.43	41.6	48.6	143.2
A9	28.9	29.02 ± 0.68	40.1	20.0	118.7
A11	32.0	35.01 ± 0.59	48.7	39.6.	155.9
A16	8	36.42 ± 1.78	42.4	53.2	170.6
A19	23.6	25.77 ± 1.13	36.6	41.8	128.9

From the rescreening results, the strains with more and stronger functions were basically consistent with the preliminary screening results, indicating that the main target traits of the screened strains were relatively stable. On the basis of the information from preliminary screening and rescreening and in accordance with the principle of functional complementarity, A3, A4, A5, A7, A8, A11, A16, and A19 were proposed as candidate strains for strain combination.

### Identification of candidate strains

**Fig 3:**
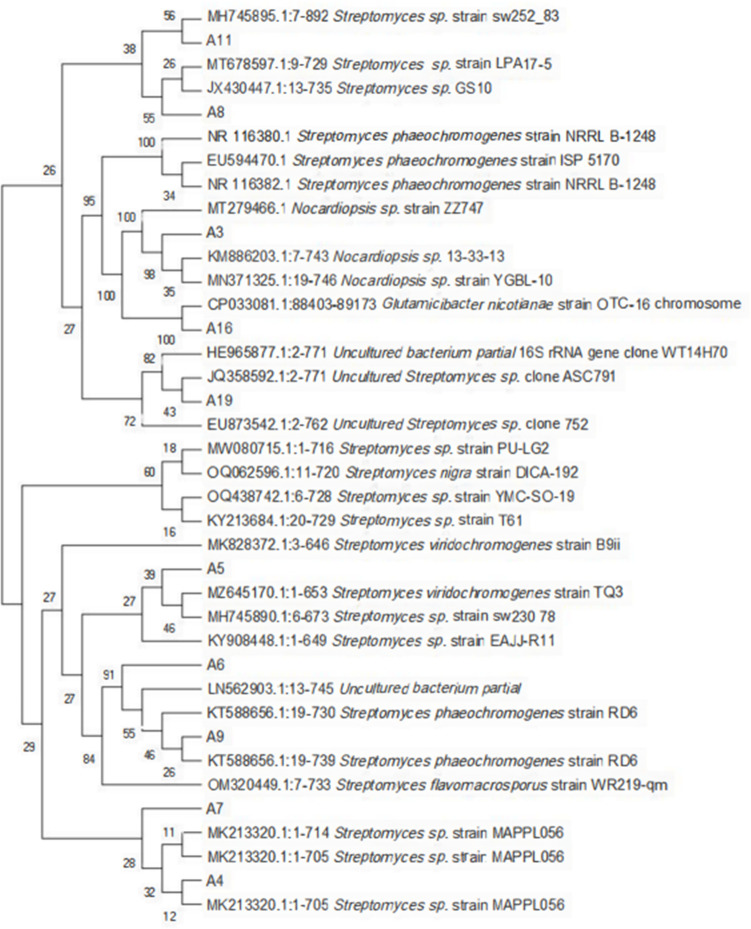
The ML phylogenetic tree based on 16S rRNA gene of the strains selected after rescreening.

**Fig 4:**
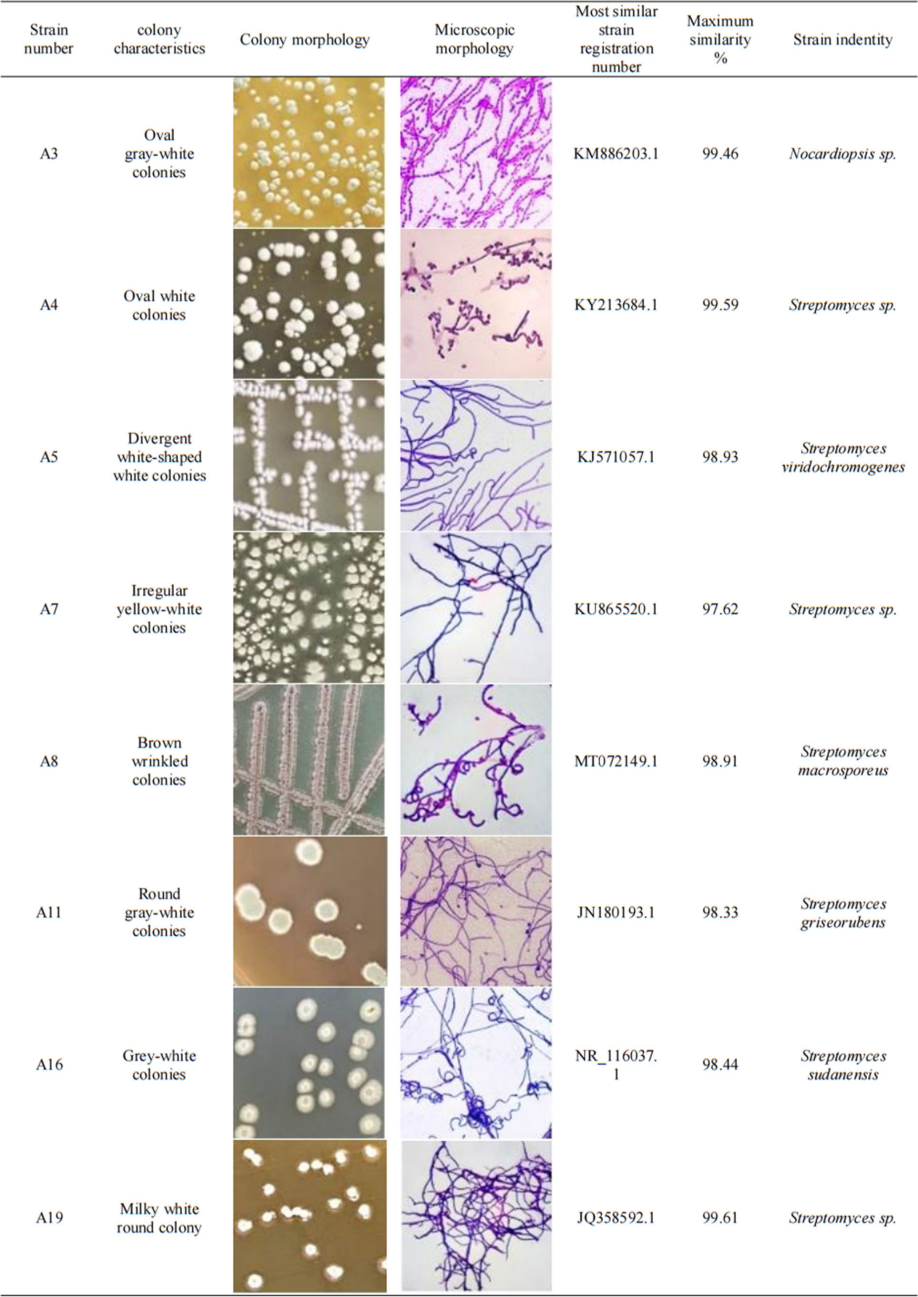
Identification results of selected strains after rescreening.

According to the culture characteristics, colony morphology, Gram staining, and microscopic observation, the strains obtained by preliminary screening were actinomycetes. From the BLAST alignment of the 16S rRNA gene sequence in NCBI, 2 of the 20 preliminary screening strains were *Nocardiopsis* sp., and the remaining 18 strains were *Streptomyces* sp. The identification results of the eight strains selected for rescreening are listed in Fig. 4. MEGA-X software was used to compare the 16S rRNA gene sequences of the selected strains with those of several related species in GenBank and to construct the maximum likelihood (ML) phylogenetic tree, as shown in Fig. 3.

Figure 4 indicates that strains A3, A4, A7, and A19 were only identified up to the genus level. Strains A5, A8, A11, and A16 were identified up to the species level, but the similarity was not high.

### Selection of strain combination

The results of the plate confrontation test showed that no obvious antagonism existed between the other strains, except that between A19 and A11 and that between A19 and A7. In consideration of the results of functional screening, rescreening, and ecological test, strains A3, A7, A8, A11, and A16 were selected to form a strain combination and prepare a complex microbial inoculant (Ac) with equal proportion of biomass.

### Degradation effect of microbial inoculant

The degradation rate and statistical analysis of straw lignocellulose in fermentation medium by A3, A7, A8, A11, A16, and their complex microbial inoculant AC are shown in Table 3.

**TABLE 3:** Comparison of degradation rate of straw lignocellulose by different strains and their complex inoculant

Strain (combination)	Degradation rate (%)				Significant difference comparison
	1	2	3	Average	
A3	32.2	30.8	35.3	32.8	bc
A7	33.1	27.3	31.8	30.7	bc
A8	28.8	25.6	30.2	28.2	c
A11	30.6	34.2	27.7	30.8	bc
A16	5	32.9	35.1	34.0	b
AC	39.9	42.7	36.7	39.8	a

Table 3 demonstrates that the degradation rate of lignocellulose from straw by AC was highest (39.8%). The difference between the complex microbial inoculant and its component strains was significant (*P* < 0.05) according to one-way ANOVA.

## DISCUSSION

Owing to the strong selectivity of the sample collection, enrichment culture, and screening methods designed in the experiment, although the total number of strains obtained from the preliminary screening was not large, the number of strains with target functions and multifunctions was not small. This series of methods improved the efficiency of isolation and screening for high-quality target strains and saved time and cost.

According to colony morphology, Gram staining, and microscopic observation, combined with the BLAST alignment of the 16 S rRNA gene sequence in NCBI, 18 of the 20 preliminary screening strains were *Streptomyces* sp., and two were *Nocardiopsis* sp. From the separation of samples in this experiment, *Streptomyces* was the dominant genus among the actinomycetes with aerobic composting-related effects, which was consistent with the results of most previous studies (22 – 24). Among the eight rescreened strains, four strains were identified as genus, and the remaining four strains were identified as species; however, the similarity was not high. CA comparison of the ML phylogenetic tree of the rescreened strains with the BLAST alignment results showed that that some branches of strains were not adjacent to those of the strains with the highest similarity. This finding suggested the possible existence of new actinomycete species in various environments that need to be continuously explored. Accurate identification of actinomycete at the species level requires not only the selection of appropriate existing methods but also the further improvement of actinomycete taxonomy and identification methods.

In this experiment, the results of Petri plate confrontation culture were used to determine the ecological relationship between two candidate strains. On the premise of eliminating the strains with obvious antagonistic effects, the strains with multifunction, strong function, and complementary function related to the aerobic composting effect were selected to form the strain combination AC. Under the same inoculation rate and fermentation conditions, the degradation rate of straw lignocellulose by the complex microbial inoculant was significantly higher than that of each single strain. This result indicated that strains of the combination were well compatible and played complementary and synergistic effects.

### Conclusions

Compost-related multifunctional actinomycetes were efficiently screened through sample collection, enrichment, separation, and a series of screening methods. In this experiment, 20 different functional actinomycetes were isolated from 56 samples, resulting in an isolation rate of 40%. Through preliminary screening and rescreening, eight strains of actinomycetes with more target functions and stronger capabilities were obtained, resulting in a screening rate of high-efficiency actinomycetes of 40%. The dominant genus of actinomycetes isolated in this experiment was *Streptomyces* (18/20), followed by *Nocardia* (2/20). An appropriate strain combination was selected by analyzing the functional assignment integral and ecological relationship. The lignocellulose degradation rate of the composite microbial agent reached 39.8%, which was significantly higher than that of single strains. The screened multifunctional actinomycete strains and their combinations enriched the actinomycete germplasm resources in microbial enhanced aerobic composting.

## Data Availability

The author confirms that all the data have been presented in the paper.
